# Association of an *INSIG2 *obesity allele with cardiovascular phenotypes is gender and age dependent

**DOI:** 10.1186/1471-2261-10-46

**Published:** 2010-09-29

**Authors:** Kimberly A Skelding, Glenn S Gerhard, Helen Vlachos, Faith Selzer, Sheryl F Kelsey, Xin Chu, Robert Erdman, David O Williams, Kevin E Kip

**Affiliations:** 1 Weis Center for Research, Geisinger Medical Center, 100 North Academy Avenue, Danville, PA 17822, USA; 2 Department of Epidemiology, University of Pittsburgh, PUBHL Pittsburgh, Pittsburgh, PA 15260, USA; 3 Division of Cardiology, Brigham and Women's Hospital, 75 Francis St, Boston, MA 02115, USA; 4 USF College of Nursing Research Center, University of South Florida, 4202 E. Fowler Avenue Tampa, FL 33620, USA

## Abstract

**Background:**

The *INSIG2 *gene has been implicated in cholesterol metabolism and a single nucleotide polymorphism (SNP) near *INSIG2 *has been shown to be associated with obesity. We sought to determine the relationship of the *INSIG2 *SNP to cardiovascular disease (CVD) related phenotypes.

**Methods and Results:**

Nine hundred forty six patients undergoing percutaneous coronary intervention (PCI) in wave 5 of the multicenter NHLBI Dynamic Registry were genotyped using RT-PCR/TaqMan/allelic discrimination for the rs7566605 SNP near the *INSIG2 *gene. Clinical variables analyzed include demographics, medical history, and procedural details. The prevalence of peripheral vascular disease (PVD) was significantly higher in older men (≥65 years) who were either homozygous or carriers of the obesity/lipid risk allele ("C") compared to non-carriers (odds ratio 3.4, p = 0.013) using a logistic regression model incorporating history of hypercholesterolemia, history of hypertension, cerebrovascular disease, history of diabetes, and BMI. A similar relationship with cerebrovascular disease was found in older (>65) women (odds ratio 3.4, p = 0.013). The *INSIG2 *SNP was not associated with BMI, nor with other clinical variables.

**Conclusion:**

Age and gender may influence the association of the *INSIG2 *obesity SNP with PVD and cerebrovascular disease in patients with pre-existing CVD.

## Background

Risk factors for atherosclerosis related disorders include dyslipidemia and obesity. The insulin signaling protein type 2 gene (*INSIG2*) has been shown to be involved in lipid and cholesterol metabolism in vitro and in animal studies [1-3], and has been linked to obesity in humans through genetic studies. For example, *INSIG2 *interacts with transcription factors that activate the synthesis of cholesterol and fatty acids in the liver and other organs [4]. In addition, a common single nucleotide polymorphism (SNP rs7566605) near the *INSIG2 *gene was found to be associated with BMI in a genome-wide association study from the Framingham Heart Study offspring cohort [5]. Playing both a key role in cholesterol homeostasis and as a genetic susceptibility factor for obesity makes it an attractive candidate locus for lipid-related phenotypes such as cardiovascular disease, as well as for BMI.

The initial association of BMI with homozygosity for the minor allele of the *INSIG2 *rs7566605 SNP was followed by both negative and positive associations involving analysis of many thousands of individuals [6-9]. Studies in severe obesity have also failed to associate the SNP with blood lipid parameters [10,11], although medication use (e.g., statins) was not accounted for. However, in populations with lower BMI levels, a gender effect may be present [12,13]. Little or no association has been found with coronary artery disease in population-based studies [10,14-16], nor with obesity in cohorts recruited for cardiovascular phenotypes [6]. The goal of this study was to determine the relationship between the *INSIG2 *rs7566605 SNP and multiple cardiovascular phenotypes in a well-characterized population of patients with known coronary artery disease.

## Methods

### Population

The National Heart Lung and Blood Institute-sponsored Dynamic Registry was utilized for this study. In brief, the multi-center Dynamic Registry was designed to characterize changes in clinical practice, particularly evolving technology, on short- and long-term patient outcomes. Five recruitment waves of approximately 2000 patients each have been enrolled and followed over the past 10 years to examine trends in PCI. Each center received approval from its institutional review board. No control patients not undergoing PCI were included in the study.

This analysis was restricted to Wave 5 patients recruited from February 2006 to August 2006 who consented to the genomic sub-study. Of the 15 Wave 5 clinical centers, 11 participated in the collection of blood samples. The study population consisted of 947 patients, 70.6% of whom were male with a median age 64 years and a median BMI of 29.0 kg/m^2^, in whom at least one stent had been placed with a yearly post-procedure follow-up scheduled to 5 years. The racial/ethnic distribution was 77.7% Caucasian, 14.0% African American, 1.1% Asian, and 7.0% Hispanic. Approximately 35.3% of patients never smoked, 24.1% were current smokers at the time of enrollment, and 40.6% were former smokers. A history of hypertension was found in 78.2%, hypercholesterolemia in 79.4%, a prior myocardial infarction in 24.8%, cerebrovascular disease in 7.0%, renal disease in 9.9%, peripheral vascular disease (PVD) in 7.5%, pulmonary disease in 8.1%, cancer in 8.5%, and diabetes in 37.4%.

Of the 947 patients in Wave 5 consented for the genomics sub study, 821 (86.7%) received only drug-eluting stents, 48 (5.1%) received bare-metal stents only, 39 (4.1%) received both a drug-eluting stent and a bare-metal stent and 39 (4.1%) received balloon angioplasty alone.

### Data

Data collected included baseline demographic, clinical, and angiographic characteristics and procedural details during the index PCI, as well as the occurrence of death, myocardial infarction, and the need for coronary-artery bypass grafting (CABG) during hospitalization. A blood draw for genomic analysis was added to Wave 5 and was obtained in dually consented patients (n = 947). Follow-up status was ascertained at 1 month, 6 months, and annually thereafter. The follow-up rate at 1 year was 97%. With the use of the Social Security Administration's Death Master File (http://www.ntis.gov/products/ssa-dmf.aspx), coordinators periodically evaluated the vital status of patients who were lost to follow-up. For patients who underwent subsequent repeat revascularization (either PCI or CABG), vessel-specific and lesion-specific data were collected whenever possible to determine the occurrence of target-vessel revascularization.

### DNA isolation

DNA was extracted from 0.35 ml of EDTA anti-coagulated whole blood using the Qiagen MagAttract DNA Blood Midi M48 Kit and Qiagen BioRobot M48 Workstation (Qiagen, Valencia, CA) according the manufacturer's directions. The final elution volume was 200 ul. Quantification of DNA extracted was performed using a NanoDrop ND-1000 spectrophotometer (NanoDrop Technologies, Wilmington, DE).

### Genotyping

Single nucleotide polymorphism (SNPs) genotyping was performed on an Applied Biosystems 7500 real-time PCR System (Applied Biosystems, Foster City, CA)[17]. Assay reagents for each SNP were obtained from Applied Biosystems (*INSIG2*, rs7566605, assay ID: C__29404113_20). DNA was genotyped according to the manufacturer's protocol. Briefly, the reaction components for each genotyping reaction were as follows: 10 ng of DNA, 5 μL of TaqMan Genotyping Master Mix (Applied Biosystems, Foster City, CA), 0.25 μL of assay mix (40×), and water up to a total volume of 10 μL. The thermocycler conditions were as follows: 50°C for 2 min, 95°C for 10 min, and 40 cycles of 95°C for15 sec and 60°C for 60 sec. The reaction was then analyzed by Applied Biosystems Sequence Detection Software (version 2.01).

### Statistical analysis

All statistical analyses were performed with the use of SAS software, version 9.1, and a two-sided P value of ≤0.05 was considered to indicate statistical significance. Differences in baseline characteristics between the groups were detected using a Chi-Square test for the categorical variables and a T-test for the continuous variables when 2 groups are present. ANOVA was used for testing differences among 3 or more groups of continuous variables. One year cumulative event rates of clinical outcomes (MI, CABG, repeat PCI, Stent Thrombosis and composite outcomes (MI/CABG, CABG/rep PCI, MI/CABG/rep PCI) were estimated by the Kaplan-Meier method and tested by the log-rank statistic. Fisher's exact test was used to test *INSIG2 *genotypes for departure from Hardy-Weinberg equilibrium using the Helix Tree software package (Golden Helix, Bozeman, MT).

Four age and gender groups were analyzed using a stepwise logistic regression model to estimate the independent effects of different variables of interest, as well as the *INSIG2 *genotype. For men >= 65 years of age, PVD was close to being significant. The odds ratio of the CC_GC genotype from the model was adjusted for history of hypercholesterolemia, history of hypertension, cerebrovascular disease, history of diabetes, and BMI. For men < 65 years of age, history of hypercholesterolemia was close to being significant. A similar analysis was used to adjust the odds ratio of the CC_GC genotype. The additional variables in the model that were adjusted for were PVD, history of hypertension, cerebrovascular disease, history of diabetes, and BMI. For women >= 65 years of age, cerebrovascular disease was close to being significant. The additional variables the model was adjusted for were: history of hypercholesterolemia, PVD, history of diabetes, and BMI. For women < 65 years of age, history of hypercholesterolemia was close to being significant. The additional variables the model was adjusted for were PVD, history of hypertension, history of cerebrovascular disease, history of diabetes, and BMI.

## Results

DNA samples were genotyped for the *INSIG2 *(rs7566605) SNP variant. Genotyping consisted of analyzing the DNA from each patient to determine whether they carried the "G" and/or "C" DNA sequences near the *INSIG2 *gene. The *INSIG2 *"C" SNP is considered the 'obesity/lipid risk' allele. The frequency of the *INSIG2 *SNP in this population is shown in Table 1 and is in good agreement with previous studies [9]. The genotype distribution did not deviate from Hardy-Weinberg equilibrium (p = 0.82). Because patients with African ancestry represent the largest single racial/ethnic sub-group (Table 2), we compared the allele frequency of our population with available data from the HapMap (http://www.hapmap.org). Very little difference in allele frequency was present among Caucasian and African populations.

**Table 1:** Frequencies of the INSIG2 SNP alleles.

Population	allele	freq	count	allele	freq	count	Total
**Caucasian**	C	0.331	515	G	0.669	1041	1556
**AA**	C	0.243	66	G	0.757	206	272
**Utah**	C	0.265	60	G	0.735	166	226
**Kenya**	C	0.275	78	G	0.725	206	284
**Nigeria**	C	0.230	52	G	0.770	174	226

**Table 2:** Frequencies of INSIG2 genotypes.

Total	CC	GC	GG
(N = 947)	(N = 94)	(N = 413)	(N = 440)
**White**	85 (10.9%)	345 (44.3%)	348 (44.7%)
**Black**	8 (5.9%)	50 (36.8%)	78 (57.4%)
**Asian**	0 (0%)	5 (50%)	5 (50%)
**Other**	1 (4.3%)	13 (56.5%)	9 (39.2%)

The diploid *INSIG2 *SNP genotypes (i.e., "CC", "GC", and "GG") were also analyzed (Table 2). The *INSIG2 *homozygous "CC" genotype was present in ~10% of the population. The *INSIG2 *heterozygous "GC" genotype was present at 43.6% and the *INSIG2 *low obesity risk "GG" was present in 46.5%. Genotype breakdown by ethnic/racial sub-group is also presented.

We then determined whether the frequencies of the more than 30 clinical parameters (Table 3) related to coronary artery disease and stent placement were different among patients with the three *INSIG2 *obesity/lipid SNP genotypes. No differences, either before or after correcting for multiple comparisons, were present in BMI across genotype groups. No significant differences were present among the genotypes for gender, race/ethnicity, or smoking status. The percentage of patients undergoing a prior stent placement or CABG, as well as the percentage with a history of in-stent restenosis or the type of stent implanted was also not different among the genotypes. Although the percentages of patients with a history of prior MI and a history of cerebrovascular disease were different prior to Bonferroni correction, they did not reach significance after correction. No differences were seen for histories of diabetes, renal disease, chest pain, PVD, pulmonary disease, cancer, hypercholesterolemia, or hypertension.

**Table 3:** Patient demographics, disease history, and INSIG2 genotypes.

Characteristic	Total (N = 947)	CC (N = 94)	GC (N = 413)	GG (N = 440)	p_value	Adjusted p_value
**Age, mean, median**	64.0, 64	64.2, 64	65.1, 65	63.0, 63	0.0571	1.0000
**Female, %**	29.4	23.4	28.6	31.4	0.2748	1.0000
**Race, %**						
**White**	77.7	87.2	80.8	72.7	0.0490	1.0000
**Black**	14.0	8.5	11.4	17.5		
**Asian**	1.1	0.0	1.2	1.1		
**Hispanic**	7.0	4.3	6.1	8.4		
**Other**	0.3	0.0	0.5	0.2		
**Height (cm), mean, median**	171.3, 173	172.9, 173	171.7, 173	170.6, 171	0.1450	1.0000
**Weight (kg), mean, median**	87.3, 85	89.1, 87	86.6, 85	87.5, 85	0.4407	1.0000
**Body mass index (kg/m2), mean, median**	29.7, 29	29.6, 29	29.4, 28	30.1, 29	0.1616	1.0000
**Smoking, %**						
**Never**	35.3	34.5	36.9	34.0	0.9078	1.0000
**Current**	24.1	23.0	23.3	25.1		
**Former**	40.6	42.5	39.7	40.9		
**Patient previously treated with a stent, %**	32.4	37.2	30.1	33.5	0.3268	1.0000
**History of in-stent restenosis, %**	23.3	26.5	22.6	23.1	0.8909	1.0000
**Stent type**						
**Coated, %**	16.0	19.1	13.1	18.0	0.1020	1.0000
**Uncoated, %**	9.6	12.8	10.7	8.0	0.2266	1.0000
**Prior CABG, %**						
**None**	81.4	79.8	80.3	82.6	0.8098	1.0000
**One**	16.8	19.1	17.5	15.8		
**More than one**	1.8	1.1	2.2	1.6		
**Acute MI**	25.2	23.4	27.5	23.4	0.4472	1.0000
**Prior MI, %**	24.9	31.5	20.3	27.8	0.0131	0.2882
**History Diabetes, %**	37.4	29.8	38.0	38.5	0.2716	1.0000
**History Cerebrovascular, %**	7.0	5.3	9.9	4.8	0.0125	0.2875
**History Renal Disease, %**	9.9	9.6	9.9	10.0	0.9902	1.0000
**History Chest Pain (angina), %**	54.3	57.4	53.4	54.5	0.7716	1.0000
**History PVD, %**	7.5	11.7	8.4	5.7	0.0851	1.0000
**History Pulmonary Disease, %**	8.1	7.4	8.9	7.5	0.7500	1.0000
**History Cancer, %**	8.5	9.6	9.9	7.1	0.3235	1.0000
**History Hypercholesterolemia, %**	79.4	81.7	75.9	82.2	0.0707	1.0000
**History Hypertension, %**	78.2	81.7	78.5	77.0	0.5931	1.0000

Further inspection of the data revealed that the highest percentage of patients with a history of prior MI were the patients homozygous for the "CC" obesity/lipid risk allele. Similarly, patients who were either "CC" homozygotes or "GC" heterozygotes had the higher percentage of a history of cerebrovascular disease. There was also a trend for patients with a history of PVD to have the C allele. A dominant model was therefore used to analyze the data by comparing patients homozygous for the non-risk genotype ("GG") versus the patients carrying at least one obesity/lipid risk "C" allele ("GC" plus "CC"). Because of the strong influence of gender and age on coronary artery disease, the data were also re-analyzed by age and gender (Table 4). In men ≥65 years of age, the percent with a history of PVD was almost three-fold higher in patients carrying one or two "C" alleles (Bonferroni adjusted p = 0.05). Conversely in women ≥65 years of age, the percent with a history of cerebrovascular disease was also higher in patients carrying one or two "C" alleles, although this was close to, but did not reach, statistical significance after correcting for multiple comparisons. These data suggest a sex-related effect of the *INSIG2 *obesity/lipid allele.

**Table 4:** Association of *INSIG2 *obesity allele and cardiovascular phenotypes by gender and age.

		Men, Age ≥ 65			
	Total	CC & GC	GG		Adjusted p-value
Characteristic	(N = 290)	(N = 171)	(N = 119)	p_value	
**Body mass index (kg/m2), mean, median**	27.7, 27	27.8, 27	27.6, 27	0.92	1.0000
**History Hypercholesterolemia, %**	82.6	82.2	83.2	0.83	1.0000
**Prior MI, %**	28.7	25.7	33.0	0.18	0.7200
**Cerebrovascular, %**	12.2	14.2	9.3	0.21	0.7200
**PVD, %**	10.5	14.2	5.1	0.01	0.0500
		**Men, Age < 65**			
	**Total**	**CC & GC**	**GG**		**Adjusted p-value**
**Characteristic**	**(N = 377)**	**(N = 195)**	**(N = 182)**	**p_value**	
**Body mass index (kg/m2), mean, median**	30.5, 30	30.2, 30	30.8, 30	0.30	0.9000
**History Hypercholesterolemia, %**	77.5	74.6	80.6	0.17	0.8500
**Prior MI, %**	25.4	22.7	28.3	0.21	0.8500
**Cerebrovascular, %**	3.5	4.1	2.7	0.46	0.9000
**PVD, %**	5.6	6.7	4.4	0.33	0.9000
		**Women, Age ≥ 65**			
**Characteristic**	**Total**	**CC & GC**	**GG**	**p_value**	**Adjusted p-value**
	**(N = 151)**	**(N = 79)**	**(N = 72)**		
**Body mass index (kg/m2), mean, median**	29.6, 29	29.5, 29	29.7, 29	0.83	1.0000
**History Hypercholesterolemia, %**	81.1	78.9	83.3	0.50	1.0000
**Prior MI, %**	22.7	20.5	25.0	0.53	1.0000
**Cerebrovascular, %**	8.0	12.8	2.8	0.02	0.1000
**PVD, %**	8.0	7.7	8.3	0.88	1.0000
		**Women, Age < 65**			
**Characteristic**	**Total**	**CC & GC**	**GG**	**p_value**	**Adjusted p-value**
	**(N = 127)**	**(N = 61)**	**(N = 66)**		
**Body mass index (kg/m2), mean, median**	32.3, 31	31.5, 30	33.1, 32	0.2105	0.8420
**History Hypercholesterolemia, %**	75.6	67.2	83.1	0.0412	0.2060
**Prior MI, %**	16.5	12.3	20.3	0.2351	0.8420
**Cerebrovascular, %**	4.8	5.2	4.5	0.8710	0.8710
**PVD, %**	5.6	3.4	7.6	0.3204	0.8420

A stepwise logistic regression was then used to estimate the independent effects of different variables, as well as the *INSIG2 *genotype, on PVD in men ≥65 years and cerebrovascular disease in women ≥65 years, as well as history of hypercholesterolemia for both women and men < 65 years of age. As shown in Table 5, the adjusted odds ratio for a history of PVD in older men who carried at least one *INSIG2 *obesity/lipid allele increased to 3.4 (p = 0.013). Similarly the adjusted odds ratio for a history of cerebrovascular disease in older women increased to 5.5 (p = 0.04). The association with genotype in the models was not significant for history of hypercholesterolemia.

**Table 5:** Logistic regression risk models for PVD, cerebrovascular disease, and hypercholesterolemia.

Characteristic (age and gender group)	Adjusted Odds Ratio (CC & GC/GG)	**95% C.I**.	P-value	Variables used in model
**PVD** **(men, age >= 65)**	3.4	1.3, 8.8	0.013	History of hypercholesterolemia, history of hypertension, cerebrovascular disease, history of diabetes, BMI
**History of Hypercholesterolemia** **(men, age < 65)**	0.7	0.4, 1.2	0.23	PVD, history of hypertension, cerebrovascular disease, history of diabetes, BMI
**Cerebrovascular disease** **(women, age >= 65)**	5.5	1.1, 27.7	0.04	History of hypercholesterolemia, PVD, history of diabetes, BMI
**History of Hypercholesterolemia** **(women, age < 65)**	0.4	0.2, 1.0	0.07	PVD, history of hypertension, cerebrovascular disease, history of diabetes, BMI

## Discussion and Conclusions

Cardiovascular disease is associated with a number of risk factors such as smoking, hypercholesterolemia, hypertension, type 2 diabetes, and abdominal obesity. Genetic variations may influence susceptibility to these risk factors, as well as direct susceptibility to CAD or MI [18,19]. Obesity is a multi-factorial process that is associated with an increased risk of cardiovascular disease, suggesting that they may share common determinants. An ideal candidate for such a shared predisposition is the *INSIG2 *gene, which may influence susceptibility through effects on cholesterol metabolism and/or obesity.

Previous studies have failed to find a significant association between the *INSIG2 *rs7566605 SNP and history of CAD/MI [10,14,15]. We cannot determine whether this is also the case for our population, which consisted of patients with existing coronary artery disease because a weakness of the study design is the lack of a control population without CAD. Given these limitations, no association was found with CAD/MI severity related phenotypes including acute MI, history of prior MI, chest pain, and prior CABG. No association of rs7566605 was found with the cardiovascular risk factors hypertension, type 2 diabetes, obesity, or smoking.

The relationship of the *INSIG2 *obesity/lipid risk allele ("C") with hyperlipidemia appears to be complex. The rs7566605 SNP has been associated with a lower prevalence of hypercholesterolemia in a study of 885 Japanese Americans (347 men and 538 women) and 378 Japanese individuals (182 men and 196 women), in which the rate of hypercholesterolemia was lower in Japanese American women homozygous for the obesity risk allele (GG, 62.2%; GC, 57.1%; CC, 42.1%), but not in the other subjects [13]. No significant differences in BMI, waist circumference, or percentage body fat were found in either population. In contrast, in a study of 2,364 Koreans, the rs7566605 SNP was found to be associated with total cholesterol levels in a dominant model in females, although this association did not meet statistical significance after performing a Bonferroni correction [12]. Again, no association was found with BMI. Our initial analysis found a lower percentage of female patients with a history of hypercholesterolemia who carried at least one copy of the *INSIG2 *obesity/lipid risk allele ("C") that was not significant after correcting multiple comparisons. However, we did not have access to actual cholesterol measurements, thus patients with any degree of hypercholesterolemia were included in the same case group. Confounding effects of lipid-lowering medication use may also be important. Future studies addressing these factors will be needed to fully assess the relationship of the *INSIG2 *SNP and cholesterol levels.

We also did not find association with BMI. However, in animal models, the effect of the *INSIG2 *gene on body weight was seen with a low cholesterol diet [4]. Diet composition was not reported or available for our study or for most studies on BMI.

Our data indicate that the *INSIG2 *obesity/lipid risk allele ("C") was associated with a higher rate of PAD in older men and an even higher rate of cerebrovascular disease in older women, suggesting significant age and gender related effects. Several other variants have also been related to PAD [20,21], with one sequence variant on chromosome 9p21 also associated with a variety of atherosclerotic phenotypes [17,19,22,23]. Relatively few data is available on the genetic predisposition to CVD [23,24]. PAD and CVD are both due to an atherosclerotic process, yet it is likely that specific molecular differences result in clinical disease in either the peripheral or cerebral vasculature. Larger studies on specific cohorts of patients with PVD and cerebrovascular disease will be needed to replicate our findings for *INSIG2*.

How the specific SNP sequences in the *INSIG2 *gene alter the function of the *INSIG2 *RNA and/or protein to increase the risk for PAD and CVD is not known. The *INSIG2 *SNP is located about 10,000 bases upstream from the coding region, so is likely to be involved in regulating the level of RNA and therefore the amount of protein produced. Future studies will be required to determine the molecular mechanism by which the specific *INSIG2 *DNA variants affect gene function.

## Competing interests

The authors declare that they have no competing interests.

## Authors' contributions

RE and XC carried out the genotyping. HV, FS, and SFK performed the statistical analysis. DOW and KEK participated in its design and coordination. KAS and GSG conceived of the study, participated in its design and coordination, and drafted the manuscript. All authors read and approved the final manuscript.

## Pre-publication history

The pre-publication history for this paper can be accessed here:

http://www.biomedcentral.com/1471-2261/10/46/prepub
